# Fire disaster preparedness and situational analysis in higher learning institutions of Tanzania

**DOI:** 10.4102/jamba.v9i1.311

**Published:** 2017-01-27

**Authors:** Jacob M. Kihila

**Affiliations:** 1 Institute of Human Settlements Studies, Ardhi University, Tanzania

## Abstract

Fire disasters are accompanied with devastating impact affecting both lives and properties. The magnitude of the impacts has been severe in places with low levels of fire disaster preparedness. A study was conducted in Dar es Salaam, Tanzania, to investigate the level of fire disaster preparedness considering the availability and condition of firefighting facilities as well as the knowledge on fire management among the selected 10 higher learning institutions. Information for the buildings was obtained from the interviews with the managers of the buildings and field observations; information on the user’s preparedness was obtained from interviews using structured questionnaire conducted with the users of the buildings including the visitors. Results from the studied buildings indicated that 60% of the firefighting facilities were not regularly serviced; 50% stored some hazardous materials; 70% of them had not enough water storage for firefighting purposes; 60% had no identifiable fire assembly points; and 90% of the sessions conducted in the buildings involved more than 100 people in a single venue. Further results indicated that 51% of the respondents were not able to operate the installed firefighting facilities; 80.7% of the respondents had never received any training on firefighting and prevention; 95.6% of the respondents had never participated in any fire drills; and 81.5% of them were not aware of the fire responder’s contacts. General situation indicated that higher learning institutions are not well prepared to manage fire outbreaks suggesting that plans to rectify the situation are imperative.

## Introduction

Disaster preparedness is one of the important elements in disaster risk reduction and it encompasses community awareness, readiness to render appropriate responses and quick recovery (Ejeta et al. 2015). Despite its importance, less has been done globally to improve the levels of disaster preparedness (Paton 2003). Little has been documented on the levels of preparedness for specific types of disasters, specially for developing countries like Tanzania. Disaster preparedness can be enhanced through strengthening the community capacities, education and improving the preventive mechanisms (Mathbor 2007). Knowledge on the levels of preparedness can inform the disaster management process and lead to well-informed plans and decisions. On the other hand, lack of disaster preparedness as it has been reported in some categories of disaster such as floods and landslides (Miceli et al. 2008), hurricanes (Howe 2011), earthquakes (Srinivas & Nakagawa 2008) and fires (Kukali & Kabuka 2009) can result in negative economic and social consequences (Wilson et al. 2007). Therefore, preparedness becomes an important aspect for achieving sustainable disaster management. Disaster preparedness in this context is defined as the measures taken to prepare for and reduce the effects of disasters. This involves prediction, where possible prevention, mitigation, appropriate responses and effective coping mechanisms against the consequences. Prevention and reduction as well as appropriate responses depend much on the public awareness and the availability and condition of supportive facilities.

Fire disaster in buildings is among the known man-made disasters with the most devastating events that cost life and properties (Shaluf 2007; Xin & Huang 2013). Globally, many recurring fire incidences have been reported (Ibe et al. 2014). Apart from loss of property and life, fire disasters have been associated with prevalence of diseases that have been reported to contribute about 1% of the global diseases burden (Leistikow et al. 2000). The most devastating effects caused by fire in buildings, as recorded in literature, include the collapse of the World Trade Centre (Cowlard et al. 2013), the fire disaster in Sweden that occurred in 1998 killing 63 people (Cassuto & Tarnow 2003), and the Mumbai and Sharjah high-rise buildings fire incidences to mention a few.

The impact of fire disasters has been very significant in high-rise buildings because most of them are not only publicly accessed but also do accommodate many people and valuable properties (Murage 2012). In addition, fire outbreaks in high-rise buildings have been more complex because these are associated with rapid fire and smoke spread, difficult evacuation procedures and firefighting as well as the possibility of accidents (Ma & Guo 2012). Therefore, special attention will be focused on public high-rise buildings in this study.

Literature highlights the importance of having a set of actions in place to ensure that fire risks in high-rise buildings are minimised. One of the actions is the enforcement of building codes that advocates, among others, provision of the facilities for firefighting to ensure compliance to all the safety requirements (Hadjisophocleous & Benichou 1999). Another action is imparting knowledge on the use of the installed facilities and awareness on the appropriate responses in case of fire outbreaks (Takao et al. 2004). Unfortunately, both actions have been underrated in most of the buildings to such an extent that when fire incidences occur, people fail to respond appropriately (Kachenje et al. 2010). Many buildings in developing countries cities are not equipped with the necessary firefighting facilities, which suggest that enforcement of building codes is still a challenge. Studies conducted in Tanzania, Kenya, Nigeria and Ghana confirm that lack of availability of facilities, poor conditions of the available facilities and lack of awareness among users are among the factors for high fire risks (Amoako 2014; Kachenje et al. 2010; Makachia et al. 2014; Sankey et al. 2014).

This article presents the findings from a study that was conducted to assess the fire disasters preparedness among users of the high-rise buildings in higher learning institutions in Dar es Salaam Tanzania. Based on previous studies, it was established that factors, such as the presence and condition of firefighting facilities and the public awareness on the use of the facilities, among others, were the important and relevant factors for the prevention of fire disasters in buildings. While the presence and condition of firefighting facilities reveals the supportive capacity of the institution, public awareness highlights the public’s ability to render appropriate responses in case of fire outbreak. Study on the human awareness to fire hazards and the evaluation of the preparedness are among the key elements for assessing fire risks (Siu 1999). Literature indicates that fire risk is based on human behaviour (Kobes et al. 2008, 2010). Other scholars highlighted the assessment of the risks and the public perceptions as an essential element for reducing the risks and that it is important to include social aspects in the provision of fire safety and management (Chow & Hung 2010; Gwynne 2008; Ibrahim et al. 2011; Zmud 2008). Therefore, this article provides important knowledge for users of high-rise buildings, specifically those in public institutions on how to improve the fire disaster preparedness. The study among other issues explored the rate of visitations to the buildings; the availability and condition of the firefighting facilities; accessibility to the available facilities; awareness on the presence and location of the escape ways; knowledge on how to operate the facilities; frequency of trainings and fire drills; and the level of awareness and the ability to appropriately respond in case of fire outbreak.

## Methodology

### Choice of the case study and sampling

The case study was conducted in Dar es Salaam, the largest City in Tanzania having a variety of mixed cultures and with an estimated population of 4.4 million people. The choice of the city was based on the highest number of training institutions and the best heterogeneous representation of the institutions. The institutions available in the city were identified and classified according to two categories, that is, being public or private and being university or college. In Tanzania, universities fall under the Tanzania Commission for Universities (TCU) and colleges fall under the National Council for Technical Education (NACTE). Non-probability sampling method was used to select a sample size of 15% from both categories (Table 1). Criteria for selecting an institution were the number of users (regular users and visitors) and the presence of multi-storey buildings. The total number of respondents was 250, whereby at least 10 permanent workers, 10 students (regular visitors for lectures) and 5 visitors (normal official visitations) were interviewed from each of the selected institution.

**TABLE 1:** Higher learning institutions in Dar es Salaam and sampling.

S/N	Category of institution	Number of institutions available in the sampling unit (sampling frame)	Size of sample
1	Public universities and colleges in Dar es Salaam	5	2
2	Private universities and colleges in Dar es Salaam	6	2
3	Public institutions under NACTE in Dar es Salaam	13	3
4	Private institutions under NACTE in Dar es Salaam	42	3

**Total**	**-**	**66**	**10**

*Source:* Tanzania Commission for Universities, NACTE

NACTE, National Council for Technical Education.

**FIGURE 1:**
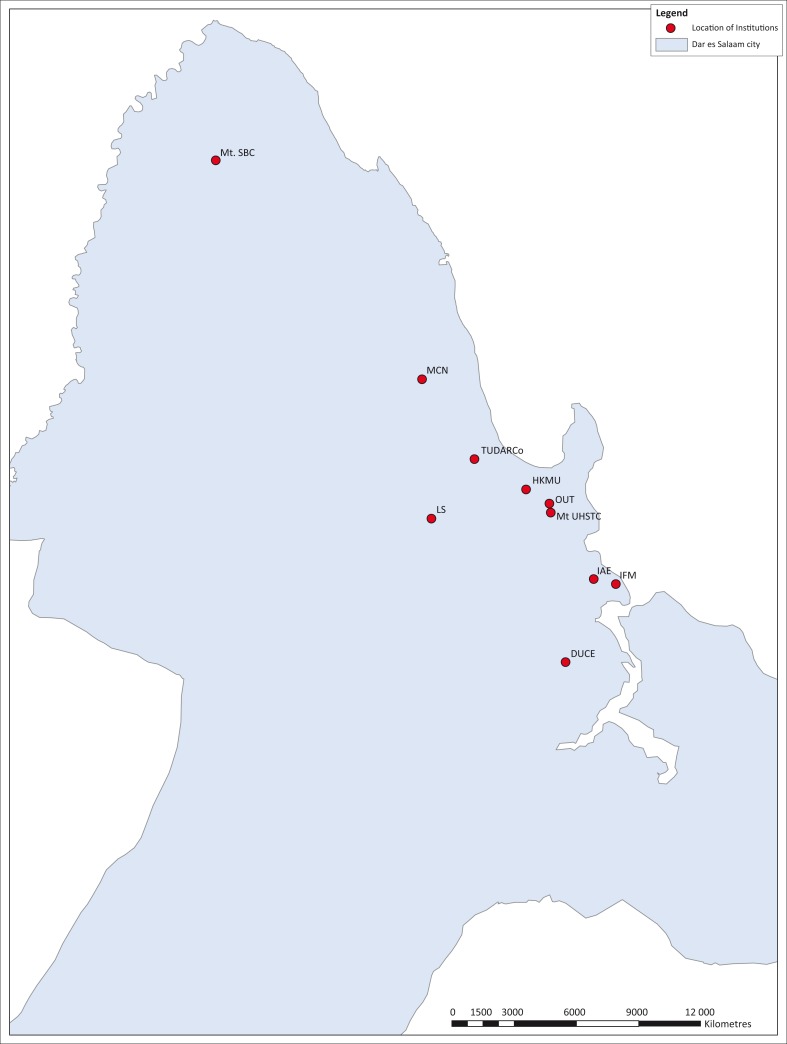
Location of the institutions under study.

Multi-storey buildings were of interest because they harbour a large number of people and properties but also the fact that whenever a fire outbreak occurs, the magnitude of the impacts in such buildings becomes high as compared to single-storey buildings. Purposive sampling was used to sample a building within the selected institutions; however, the criteria for sampling in this case were the number of storeys and users. Selection of the interviewees (users of the building) was done using the random sampling method while that of the key informants for the sampled building was done purposively to include the Estate Managers or Heads of the Institutions. Locations and the details of the selected institutions are as shown in Figure 1 and Table 2.

**TABLE 2:** Institution sampled for the study.

S/N	Selected institutions	Type of institution	Number of buildings with multi-storey
1	Dar es Salaam University College of Education (DUCE)	Public university	11
2	Open University (OUT)	Public university	6
3	Hubert Kairuki Memorial University HKMU)	Private university	2
4	Tumaini University (TURDARCo)	Private university	1
5	Institute of Finance Management (IFM)	Public institutions under NACTE	4
6	Law School of Tanzania (LS)	Public institution under NACTE	3
7	Institute of Adult Education (IAE)	Public institution under NACTE	1
8	Massana College of Nursing (MCN)	-	1
9	Mt. Sinai Business College (Mt. SBC)	Private institutions under NACTE	3
10	Mt Ukombozi Health Sciences Training Centre (Mt. UHSTC)	Private institution	1

NACTE, National Council for Technical Education.

### Data collection methods and analysis

Data were collected using structured questionnaires administered to different users of the buildings including the owners and managers of the buildings, workers and students as well as visitors to the buildings. Field observations accompanied by photographic registration were employed to substantiate the information collected. Positions of the institutions were recorded using Geographical Positioning System handset and the information was handled in the GIS environment using the ArcGIS 10 software. Data analysis was done using SPSS software, and the information was correlated with previous related studies.

## Results and discussion

### Information about the buildings

All the selected buildings were multi-storey with the number of storeys ranging from 4 to 11 (Table 3). Further details indicate that the oldest buildings are the Hubert Kairuki Memorial University (HKMU), Institute of Adult Education (IAE), Institute of Finance Management (IFM) and Massana College of Nursing (MCN) while the most recent are Tumaini University (TUDARCo), Dar es Salaam University College of Education (DUCE), Open University of Tanzania (OUT) and Mt. Sinai Business College (Mt. SBC). The findings show that there were a significant numbers of users and visitors in all the buildings, which implies that in the case of an event of fire outbreak, adverse impact may occur. Almost all the sampled buildings (70%) had some firefighting water storage facilities. The presence of firefighting water storage facilities suggests compliance with building requirements for fire responding.

**TABLE 3:** Information about the selected buildings.

S/N	Institution	Name of building	Number of stories	Use of building	Year of operation	Average number of users	Average number of visitors	Firefighting water storage (m^3^)	Firefighting facilities available
1	DUCE	Teachers Professional Centre (TPC)	4	Office and lectures	2014	60		20	FE, HR, FA and SFS
2	OUT	Open and Distance Learning (ODL) Tower	10	Multi-use	2013	150	70	15	FE, HR and FA
3	HKMU	Kairuki Hospital	10	Multi-use	1987	250	100	24	FE and HR
4	TURDARCo	New Tower	11	Multi-use	2015	2160	60	48	FE, HR, and FA
5	IFM	Block D	6	Multi-use	1992	520	100	40	FE and HR
6	LS	Teaching Block	4	Office and lectures	2012	750	25	10	HR and FA
7	IAE	IAE Block	4	Multi-use	1975	500	100	1	FE, HR and FA
8	MCN	Masana Hospital	4	Multi-use	1995	250	120	-	FE
9	Mt. SBC	Teachers Education Department	4	Multi-use	2012	100		-	FE
10	Mt. UHSTC	Mt. Ukombozi Building	4	Multi-use	2009	200	50	-	FE

FE, Fire extinguisher; HR, Horse reel; SFS, Sprinkler fire system; FA, Fire detection and alarm system; DUCE, Dar es Salaam University College of Education; OUT, Open University of Tanzania; HKMU, Hurbert Kairuki Memorial University; TURDARCo, Tumani University Dar es Salaam College; IFM, Institute of Finance Management; LS, Law School; IAE, Institute of Adult Education; MCN, Massana College of Nursing; Mt. SBC, Mt. Sinai Business College; Mt. UHSTC, Mt Ukombozi Health Science Training Centre.

### Category of respondents

It was established that more than 90% of the respondents were the main occupants (students and workers) of the buildings and the rest were visitors who occasionally pay a visit to the buildings (Table 4). Of the respondents, 51.4% were female and 48.6% male respondents. The duration of time for which the users have been using the building was 48.2% within 0–1 year, 37.3% within 2–4 years and 13.3% within 5–10 years. This shows that a significant number of users were relatively new to the building. It also highlights the importance of regular schedule of trainings to capture the new users because learning institutions admit new students each year.

**TABLE 4:** The number and type of respondents from each institution type.

Type of institution	Category of respondent			Total

	Student	Worker	Visitor	
Public university	21	19	10	50
Private university	21	19	10	50
Public institution under NACTE	31	32	12	75
Private institution under NACTE	37	28	10	75

**Total**	**131**	**98**	**42**	**250**

NACTE, National Council for Technical Education.

### Available firefighting facilities and their condition

All the buildings were equipped with fire extinguishers and several others had more than one type of firefighting facilities (Table 3). The additional observed firefighting facilities include the horse-reel system, detection and alarm system and sprinkler fire system. However, it was learnt that, despite their presence, the installed facilities from 30% of the institutions were non-functioning (Table 5). Also, firefighting facilities from 60% of the institutions were not serviced, which means that despite their presence they would not deliver the intended function and hence be ineffective for firefighting. This observation may be associated with lack of awareness or the lack of commitment among the managers of the buildings. Absence or lack of maintenance of the installed facilities is considered to be one of the major obstacles to fire prevention; therefore, institutions lacking on this aspect are highly vulnerable to fire hazards.

**TABLE 5:** Fire hazard indicators for building.

Institution/building	More people than design capacity		Presence of non-functioning facilities		Facilities not serviced		Presence of hazardous materials or activities		More than 100 people gather in a common venue		Enough firefighting water stored		Building access points for fire service easily identified		Door for public lectures opens outward		Fire assembly points present and easily accessed	
								
	Yes	No	Yes	No	Yes	No	Yes	No	Yes	No	Yes	No	Yes	No	Yes	No	Yes	No
TURDARCo-New Tower	x	-	-	x	-	x	-	x	x	-	x	-	x	-	x	-	-	x
Mt. SBCEducation Department Building	-	x	-	x	x	-	x	-	x	-	-	x	x	-	-	x	x	-
Mt. UHSTC-Building	-	x	-	x	-	x	x	-	x	-	-	x	-	x	x	-	-	x
OUTODL Tower	-	x	x	-	x	-	-	x	x	-	-	x	x	-	-	x	x	-
LS-Teaching block	-	x	-	x	x	-	-	x	x	-	-	x	-	x	x	-	-	x
MCN-Hospital Building	x	-	-	x	-	x	x	-	x	-	-	x	-	x	x	-	-	x
HKMU- Hospital Building	-	x	x	-	x	-	x	-	x	-	x	-	-	x	x	-	-	x
IAEBuilding	x	-	x	-	x	-	x	-	x	-	-	x	x	-	x	-	x	-
DUCE- Teachers Professional Centre building	-	x	-	x	x	-	-	x	-	x	-	x	-	x	x	-	-	x
IFM-Block D	x	-	-	x	-	x	-	x	x	-	x	-	x	-	-	x	x	-

TURDARCo, Tumani University Dar es Salaam College; Mt. SBC, Mt. Sinai Business College; Mt. UHSTC, Mt Ukombozi Health Science Training Centre; OUT, Open University of Tanzania; ODL, Open and Distance Learning; LS, Law School; MCN, Massana College of Nursing; HKMU, Hurbert Kairuki Memorial University; IAE, Institute of Adult Education; DUCE, Dar es Salaam University College of Education; IFM, Institute of Finance Management.

Accessibility among other factors has been identified as the most important factor that can affect fire preparedness (Márquez Sierra et al. 2012). A firefighting facility that is inaccessible or difficult to access does not offer good opportunity for emergency actions. For instance, in case of flaring and raging fire, an individual may be obstructed from reaching the facility. On this issue, the study looked at the distance from the users to the nearest firefighting facility and found that most of the facilities were within the recommended 10 m from the user’s desk or station (48.2% of respondents were within 0 m – 5 m, 30.9% within 6 m – 10 m and the rest above 10 m). Firefighting facilities located more than 10 m away from the working desk or station make it difficult to access and are hence unreliable in the time of emergency.

**FIGURE 2:**
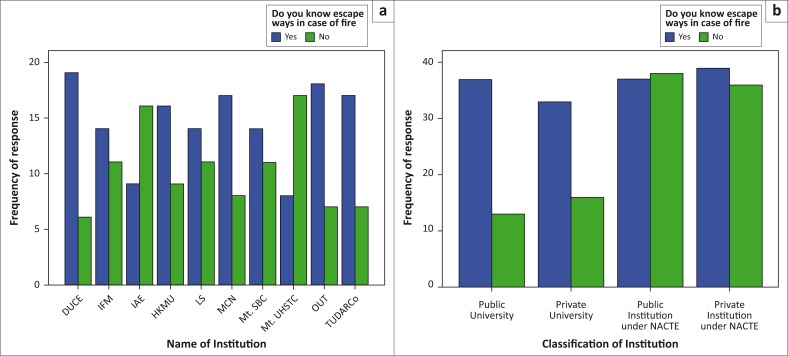
Knowledge on the location of the escape ways. (a) Responses per institution and (b) responses per institution category.

In terms of the awareness on the presence and position of the escape ways for use in case of outbreak, it was found that 58.6% of the respondents knew the positions and 41.4% of the respondents were not aware of the positions. Comparing the institutions from the two categories under study, it was observed that institutions under NACTE had relatively higher proportion of individuals who are unaware of the presence and position of the escape ways as compared to universities (Figure 2). Field observations clearly established that even in buildings where the escape ways are seen, some individuals would still not be able to locate their positions, implying that the main problem was lack of awareness or negligence. Because the presence of the escape ways and the public awareness on their positions are mentioned as important elements for safety of the users of the building, failure to identify those positions is therefore one of the risky factors (Botchway & Boatemaa-Oti 2012).

### Knowledge on how to operate the installed facilities

**FIGURE 3:**
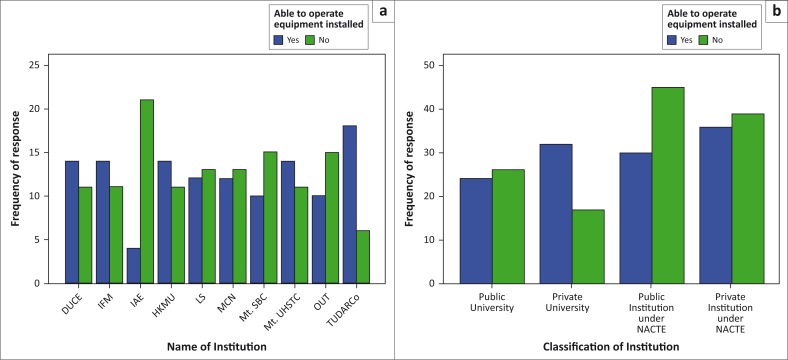
Knowledge on the use of the installed fire facilities in the selected institutions. (a) Responses per institution and (b) responses per institution category.

The study explored the ability of individuals to operate the installed facilities and found that 51% of all the respondents were not able to operate the installed equipment, while the rest were able to. In comparison, institutions such as IAE, OUT, Mt. SBC had more people without the knowledge on how to operate the facilities than the rest. This implies that the users of buildings in those institutions have low capacity to control and manage fire outbreaks as compared to the rest of the institutions (Figure 3). It was also found that public institutions had many more people who were not able to operate the installed facilities than the private institutions (Figure 3), which implies that public institutions have high fire risk as compared to the private institutions. Lack of training and orientation on fire safety in the buildings was pointed out (92.7% of respondents) as the reason for lack of knowledge.

### Training and fire drills

**FIGURE 4:**
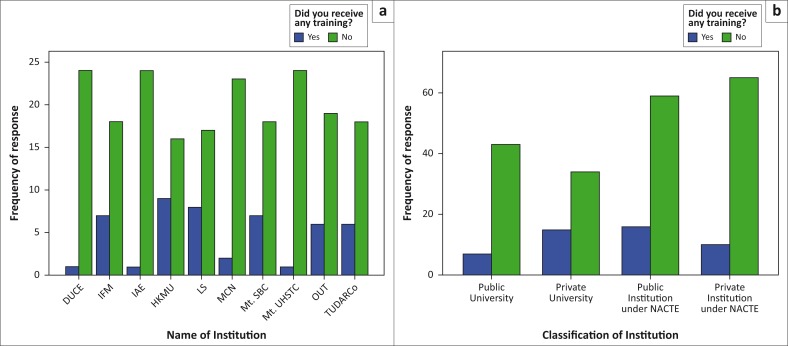
Level of training on fire preparedness in higher learning institutions. (a) Responses per institution and (b) responses per institution category.

The study revealed that training was not regularly conducted among the institutions. Of the respondents, 80.7% had never received any sort of training on how to operate the fire equipment and the basics for firefighting, while only 19.3% had received some training. Among those who were trained, about 76.6% of them had received such training more than a year ago; most of the trained were from private universities and institutions. Generally, lack of regular training to users of their buildings on fire safety was reported in almost all institutions under study, with public universities and private institutions under NACTE being the worst off (Figure 4). Similar finding was recorded in Kenya, where more than 74% of the local university employees were reported to have never received any training (Makachia et al. 2014).

Likewise, findings on the presence of fire drills were not good. Despite the fact that an effective fire preparedness programme requires fire drills be conducted after a certain time interval, findings from the study revealed that 95.6% of the respondents had never participated in any fire drill. This implies that fire drills are not part and parcel of the safety programmes for the institutions.

### Assistance and response in events of fire outbreak

**FIGURE 5:**
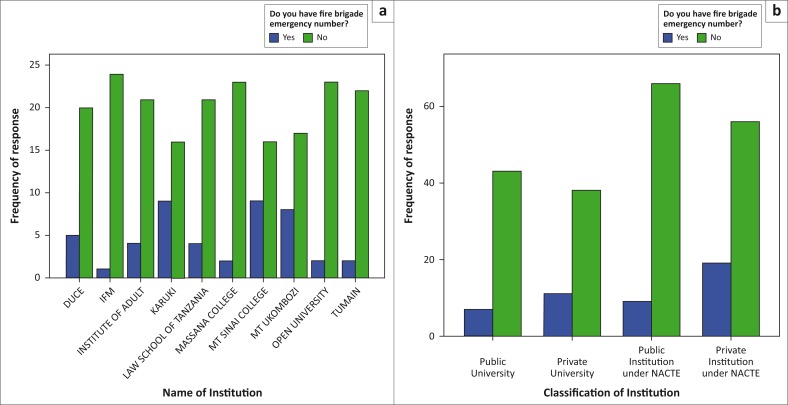
Knowledge on the fire brigade contacts. (a) Responses per institution and (b) responses per institution category.

Another important element that was investigated is the knowledge of users of the building on whether they would seek appropriate assistance in case of fire outbreak. Results showed that about 81.5% of the respondents had no contact details of fire brigade or any other fire outbreak responders whose help can be called on in the event of a fire outbreak. Comparison between the public and private institutions shows that private institutions had more people with the contact details of fire responders than the public (Figure 5). Similar findings were recorded in a study conducted in Nigeria, where more than 85% of the interviewed students had no phone number for the fire service office (Sankey et al. 2014). No or delayed communication can always be experienced in case of fire outbreak without having in place the contacts of the fire responders, a situation that would magnify the impact of fire disasters.

Human behaviour in case of fire outbreaks has been identified as one of the important elements for fire disaster management (Kobes et al. 2010). Human-first response in case of outbreak was explored and results showed that 63.1% of the respondents would react by running away in case of fire outbreak and 22.1% would seek assistance from fire brigade. This implies that, in an event of fire outbreak, confusing mixed reactions would be experienced that would lead to disruption of the rescue and response operations. Because behaviour can be tamed through training and awareness sessions, people need to be enlightened and prepared especially through fire drills to be able to act appropriately in case of fire outbreak.

### Fire hazard situational analysis

Situational analysis of the buildings revealed that most of the buildings lack the necessary means and facilities for firefighting. For example, it was evident that apart from 40% of the buildings being occupied with a greater number of people than the designed capacities, 30% of the buildings had non-functioning firefighting facilities. Furthermore, 60% of the buildings had facilities that were not serviced; 50% of the buildings did store some hazardous materials; 90% of the buildings had sessions that gather more than 100 people in a single venue; 70% of the buildings did not have enough water for firefighting; 50% of the buildings had access points for fire service that were not easily identifiable; and 60% of the buildings had no identifiable fire assembly points (Table 5). This suggests that fire preparedness is given low priority in many high-learning institutions. Installation of the basic firefighting facilities is a requirement by the existing legislation and building codes that requires developers to ensure that all the buildings are equipped with firefighting facilities. The observed situation indicates that there is non-adherence and negligence to comply with the regulations. Factors for negligence need to be further studied. Knowledge on such underlying factors can supplement the information obtained from this study and be used for setting proper fire safety management strategy.

## Recommendations

Based on the findings, fire disasters management for high-rise buildings is a challenge. To ensure that the situation is improved, the author recommends that there should be some mechanisms to ensure strict adherence to the building codes and the fire safety provisions by managers of the high-rise buildings. To arrive at this, responsible government bodies can strengthen the enforcement of laws and improve the monitoring framework. Among several other elements, the following should be given enough emphasis:

- Establishment and implementation of regular maintenance schedules to ensure that the firefighting facilities do function all the time.
- Design and implementation of regular training programmes on fire safety and prevention to ensure that the knowledge and awareness levels of building users are improved.
- Firefighting basics should be integrated in the training curriculums of the institutions and be delivered at the beginning of teaching programme to focus on new entrants.
- Induction programmes to new staff should include fire safety training among others to increase the knowledge and awareness of the new staff on fire prevention and management.

## Conclusion

Fire preparedness in terms of presence of necessary firefighting facilities, knowledge and awareness among the users of the buildings for the higher learning institutions has not received its paramount importance. It has been clearly shown that higher learning institutions in Tanzania are generally not well prepared for fire outbreaks. While lack of adequate provision and maintenance of firefighting equipment or facilities have been spotted, the lack of knowledge and skills among users of the building to use and offer appropriate response in case of fire outbreaks have been also highlighted. Analysis of the situation in terms of fire preparedness reveals that most institutions are at a high risk for fire. Despite the fact that the underlying factors for the situation are not well known, lack of commitment among the managers of institutions and buildings in ensuring that fire outbreaks are prevented and well managed is one of the suggested factors. However, more research is needed to identify the actual underlying factors and the appropriate interventions. Considering the importance and contribution of the higher learning institutions to local and international development, the identified shortcomings should be appropriately addressed by the responsible parties based on the recommendations given.
